# An Update on Gene Therapy for Inherited Retinal Dystrophy: Experience in Leber Congenital Amaurosis Clinical Trials

**DOI:** 10.3390/ijms22094534

**Published:** 2021-04-26

**Authors:** Wei Chiu, Ting-Yi Lin, Yun-Chia Chang, Henkie Isahwan-Ahmad Mulyadi Lai, Shen-Che Lin, Chun Ma, Aliaksandr A. Yarmishyn, Shiuan-Chen Lin, Kao-Jung Chang, Yu-Bai Chou, Chih-Chien Hsu, Tai-Chi Lin, Shih-Jen Chen, Yueh Chien, Yi-Ping Yang, De-Kuang Hwang

**Affiliations:** 1 School of Medicine, National Yang Ming Chiao Tung University, Taipei 11221, Taiwan; alv320429@gmail.com (W.C.); sjlin841012@gmail.com (S.-C.L.); chelsea30127@gmail.com (S.-C.L.); michaelchang1109@gmail.com (K.-J.C.); suddenonset@gmail.com (Y.-B.C.); chihchienym@gmail.com (C.-C.H.); 2 Department of Medical Research, Taipei Veterans General Hospital, Taipei 11217, Taiwan; lintingyi2014@gmail.com (T.-Y.L.); henkie.lai@gmail.com (H.I.-A.M.L.); joseph7758758@gmail.com (C.M.); km@gmail.com (A.A.Y.); taichilin0331@gmail.com (T.-C.L.); Sjchen96@gmail.com (S.-J.C.); 3 School of Medicine, Kaohsiung Medical University, Kaohsiung 80708, Taiwan; 4 Department of Ophthalmology, Taipei Veterans General Hospital, Taipei 11217, Taiwan; johnny19890821@gmail.com; 5 Institute of Pharmacology, College of Medicine, National Yang Ming Chiao Tung University, Taipei 11221, Taiwan; 6 Department of Medicine, National Taiwan University, Taipei 10617, Taiwan; 7 Institute of Clinical Medicine, National Yang Ming Chiao Tung University, Taipei 11221, Taiwan; 8 Division of Basic Research, Department of Medical Research, Taipei Veterans General Hospital, Taipei 11217, Taiwan; 9 Institute of Food Safety and Health Risk Assessment, National Yang Ming Chiao Tung University, Taipei 11221, Taiwan

**Keywords:** inherited retinal dystrophy, Leber Congenital Amaurosis, gene augmentation therapy, RNA-based antisense oligonucleotide therapy, gene-editing therapy

## Abstract

Inherited retinal dystrophies (IRDs) are a group of rare eye diseases caused by gene mutations that result in the degradation of cone and rod photoreceptors or the retinal pigment epithelium. Retinal degradation progress is often irreversible, with clinical manifestations including color or night blindness, peripheral visual defects and subsequent vision loss. Thus, gene therapies that restore functional retinal proteins by either replenishing unmutated genes or truncating mutated genes are needed. Coincidentally, the eye’s accessibility and immune-privileged status along with major advances in gene identification and gene delivery systems heralded gene therapies for IRDs. Among these clinical trials, voretigene neparvovec-rzyl (Luxturna), an adeno-associated virus vector-based gene therapy drug, was approved by the FDA for treating patients with confirmed biallelic *RPE65* mutation-associated Leber Congenital Amaurosis (LCA) in 2017. This review includes current IRD gene therapy clinical trials and further summarizes preclinical studies and therapeutic strategies for LCA, including adeno-associated virus-based gene augmentation therapy, 11-cis-retinal replacement, RNA-based antisense oligonucleotide therapy and CRISPR-Cas9 gene-editing therapy. Understanding the gene therapy development for LCA may accelerate and predict the potential hurdles of future therapeutics translation. It may also serve as the template for the research and development of treatment for other IRDs.

## 1. Introduction

Inherited retinal dystrophies (IRDs) encompass various eye diseases. Patients affected with inherited retinitis pigmentosa (RP) and choroideremia (CHM) often experience gradual loss of night vision and may also develop tunnel vision [1,2]. Others, with Leber congenital amaurosis (LCA), may be born with or experience the early onset of vision loss [3]. Patients with X-linked juvenile retinoschisis (XLRS) are commonly observed to present early-onset macular degeneration, loss in visual acuity and the splitting of retinal layers [4]. The irreversible progress of visual impairment and lack of effective treatments highlight the need for innovative therapeutic strategies. Due to the immune-privileged characteristics of eyes and the continuous identification of causative genes, gene therapy may hold great promises for treating IRDs. This review provides an overview of retinal gene therapy development by summarizing significant contributions and important clinical trials to date (Table 1).

## 2. Current Developments in Retinal Gene Therapy

### 2.1. Technological Breakthrough

Advances in the identification of causative genes inextricably propel the progress in retinal gene therapy. The identification of crucial driver genes of disease onset/progression provides significant insights into the disease mechanisms and creates the possibility of targeting these genes therapeutically. The delineation of specific underlying genomic and genetic factors in molecular diagnosis establishes the basis for gene augmentation and editing therapeutic designs. Currently, researchers have identified 260 driver genes for IRDs [18]. Recent advances in manipulating genetic material and the accumulation of several tools to tailor genetic elements gave gene therapy momentum as a promising upcoming field.

### 2.2. Retina, the Ideal Playground for Gene Therapy

A nonrenewable part of the central nervous system, the retina, is a desirable target for gene therapy due to the complex innate visual signal transmission that modernized tools cannot restore. Moreover, the eye’s unique nature promotes the appropriateness and advantages of gene therapy application for retinal diseases [19], which foster intense exploration and innovation. The retinal cells do not reproduce after birth; therefore, a single dose of the vector can ensure long-term expression of the transgene [20]. Furthermore, the eye is naturally small and easily approachable with intravitreal and subretinal injections. Its small volume allows low quantities of vectors or cells to reach the optimal concentration. Since the ocular compartment is isolated, significant extraocular leakage is rare, which lessens the risks and complexities. The blood–retina barrier assures the immune privilege status, making foreign antigens and viral vector exposure tolerable; additionally, the barrier also provides the antigen-specific inhibition of both cellular and humoral immune responses [21].

### 2.3. Gene Delivery Systems

Despite the continuous discovery of novel IRD driver genes, the translation of virus-mediated gene delivery into the clinic faces several hurdles. Due to the very nature of gene delivery with integrating vectors, translation/application/viability of gene therapy was limited by constitutive/ inherent elicitation of the immune response, insertional mutagenesis, viral tropism, off-target activity and undesired/high profile adverse clinical events [22,23]. Despite being a potentially powerful tool for correcting a disease at the genetic level, the inability to accomplish controlled manipulation and safe transgene insertion limited real-life applications of gene therapy. Therefore, the identification of safe and convenient vectors to address these concerns was critical in gene therapy advancement. Currently, gene therapy has diverged into virus-mediated and physical mechanism-based (including application of nanomaterials) gene delivery approaches. Viral vectors are primarily represented by adenovirus (AV), lentivirus and adeno-associated virus (AAV). As adenovirus vectors are immunogenic and lentivirus vectors integrate into the genome, AAV vectors are the most preferred choice for practical use. Integration into the genome may be influenced by the local chromatin structure at the targeted genome site and affect the transgene and native neighboring genes’ expression. Wild type AAVs are replication-incompetent viruses that require helper AVs to enter the lytic cycle, moreover, Rep protein supplied in trans eliminates the ability of genomic integration. The recombinant adeno-associated virus (rAAV) genome is processed into a double-stranded circular episome that is maintained extrachromosomally. The chromatin-like structure of the AAV vector genome elicits no negative effects to host cells and enables long-term transgene expression in non-dividing cells [24]. The property of cell-selective targeting based on a serotype and low immunogenicity made AAVs a real workhorse of gene therapy [19]. Furthermore, pseudotyping, a process of creating virus hybrids, is now used to further exploit/refine the tropism and transduction efficiency of each serotype [25,26,27].

## 3. Leber Congenital Amaurosis

### 3.1. Clinical Characteristics and Genetics

Leber Congenital Amaurosis (LCA) is a rare IRD with a worldwide prevalence of 1/30,000 to 1/81,000 of newborn babies, which accounts for ≥5% of all IRDs [28]. LCA patients may often experience profound vision loss, pendular nystagmus and severe retinal degeneration in infancy or early childhood.

LCA comprises a genetically heterogeneous group of mainly autosomal-recessive retinopathies beginning in infancy and childhood, with at least 21 mutated genes and over 400 mutations identified to date [29]. These genes regulate such processes as intraphotoreceptor ciliary transport (*CEP290, IQCB1, LCA5, RPGRIP1, SPATA7, TULP1, IFT140*), photoreceptor morphogenesis (*CRB1, CRX, GDF6, CLUAP1, PRPH2*), phototransduction (*AIPL1, GUCY2D, RD3*), retinoid cycle (*LRAT, RDH12, RPE65*), signal transduction (*CABP4, KCNJ13*), retinal differentiation (*OTX2*), guanine synthesis (*IMPDH1*), outer segment phagocytosis (*MERTK*), coenzyme NAD biosynthesis (*NMNAT1*), subcomponent of a chaperon complex (*CCT2*) or other protein unknown function (*DTHD1*) [29,30].

Due to the wide range of genotypic variability, the clinical phenotypes in carriers of LCA mutations are also highly heterogeneous. Variable ophthalmic disorders range from essentially normal to those characterized by refractive errors, photoaversion, nyctalopia, oculodigital reflex, peripheral chorioretinal atrophy, intraretinal pigment migration, drusen-like deposits, keratoconus and cataracts. Some LCA cases even show neurologic, intellectual, or psychomotor developmental delays [31,32,33,34,35]. In total, there are 18 recognized types of LCA, among them, LCA type 2 (LCA2) is caused by the mutation in the *RPE65* gene on chromosome 1p31. Mutations in this gene also cause retinitis pigmentosa. On the other hand, LCA10 is caused by mutations in the *CEP290* gene on chromosome 12q21. Both homozygous and compound heterozygous mutations of these genes were found to cause LCA2 and LCA10 [36].

### 3.2. Disease Mechanisms: Interdependence of Photoreceptors and RPE

The end stages of IRDs are generally characterized by irreversible progressive thinning of the retina with the loss of RPE and neurosensory retinal cells that lead to severe visual dysfunction or total blindness. Severe perturbation of gross retinal structure mainly involves the retina’s thinning upon photoreceptor cell death [37]. Moreover, IRDs also cause structural changes, such as rosette-like structure formation in retinitis pigmentosa [38] and the physical separation of cells at the outer plexiform layer in retinoschisis [37].

The pathophysiological mechanisms of LCA are related to the disruption of phototransduction and visual cycle and affect the complex homeostatic interplay between photoreceptors and the retinal pigment epithelium (RPE) layer. RPE underlies the retina and serves as an immune-privileged structure that separates the photoreceptor layer and choroidal vasculature. Therefore, the role of RPE as a physical barrier is in maintaining photoreceptor viability by providing nutrients from the choroidal blood supply and transporting away waste products from the photoreceptors [39]. Photoreceptors are specialized neurons containing the outer segments (OS) composed of stacked membranous disc carrying phototransduction proteins, opsins, which are responsible for converting captured light to electric signals. RPE phagocytoses 10% of OS discs daily and renews a complete OS in 10–15 days [40].

The visual cycle’s physiology depends on the photoreceptor’s integrity and functions, as well as on the supporting RPE cells. Therefore, immune-mediated and oxidative stress-mediated RPE damage and malfunction may lead to atrophic age-related macular degeneration with progressive loss of photoreceptors and choroid instability. RPE degeneration induced by the mutations affecting photoreceptor OS in Stargardt’s macular dystrophy emphasizes the interdependence between these two retinal layers in maintaining their structural integrity [8].

### 3.3. Visual Cycle and the Role of RPE65

**Figure 1:**
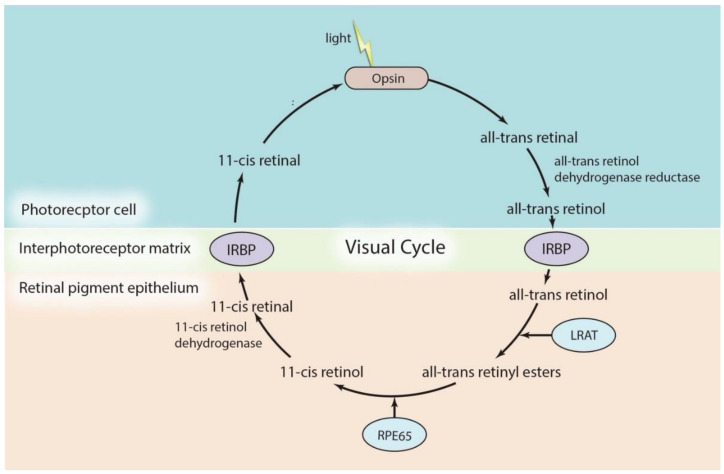
Outline of retinoid metabolism in the visual cycle. The activation of opsin by light triggers a series of conversions starting from 11-cis-retinal to all-trans-retinal. The cycle is completed by recombination of 11-cis-retinal with opsin protein to form the photoactive pigment. IRBP: interphotoreceptor retinoid-binding protein; LRAT: lecithin retinol acyltransferase; RPE65: Retinal pigmented epithelium-specific protein with molecular mass 65 kDa.

The visual cycle is initiated by the light activation of visual pigment opsin in the photoreceptor cells (Figure 1). Opsins are G protein-coupled receptors (GPCRs) that contain the covalently attached cofactor, 11-cis-retinal. The single photon of light results in the conversion of 11-cis-retinal to all-trans-retinal, which leads to a change in the conformation of the opsin’s protein moiety. The latter triggers a signal transduction cascade resulting in the closure of cyclic GMP-gated cation channels, which changes the membrane potential, generating nerve impulses that are transduced to the central nervous system to generate vision. On the other hand, all-trans-retinal is reduced into all-trans-retinol and diffuses into adjacent RPE through the interphotoreceptor matrix. The transfer of all-trans-retinol into RPE is mediated by the interphotoreceptor retinoid-binding protein (IRBP). In RPE cells, all-trans-retinol is esterified into all-trans-retinyl esters by lecithin retinol acyltransferase (LRAT). All-trans-retinyl esters are converted to 11-cis-retinol by RPE65, followed by the oxidization of 11-cis-retinol to 11-cis-retinal. This 11-cis-retinal then diffuses back to the OS of photoreceptor cells, where it is recombined with the opsin polypeptide to form photoactive pigment to complete the cycle [41,42,43].

RPE65 is a 65-kD protein specifically expressed in the RPE, which possesses isomerohydrolase enzymatic activity that is required to regenerate 11-cis-retinal [38,44,45]. The *RPE65* gene contains fourteen coding exons spanning 20 kb. In contrast to other genes whose defects also play a contributing role in degenerative retinopathies, *RPE65* is among the genes expressed exclusively in the RPE [44,45]. *RPE65* mutations interrupt the regular retinoid cycle, resulting in the absence of light-sensitive visual pigment in photoreceptors and blindness.

## 4. Preclinical Studies of the *RPE65* Gene Therapy of LCA

The ultimate goal of preclinical efforts is to achieve gene delivery to a group of heterogeneous patients who may present with different LCA onset and retinal degeneration status. The non-degenerated retina is defined as one that suffers from metabolic dysfunction only, whereby the interrupted retinoid cycle may be restored by the wild type *RPE65* gene replacement. In contrast, the degenerated retina is characterized by the loss of photoreceptors that may benefit more from visual prosthetics or stem cell therapeutics.

**Figure 2:**
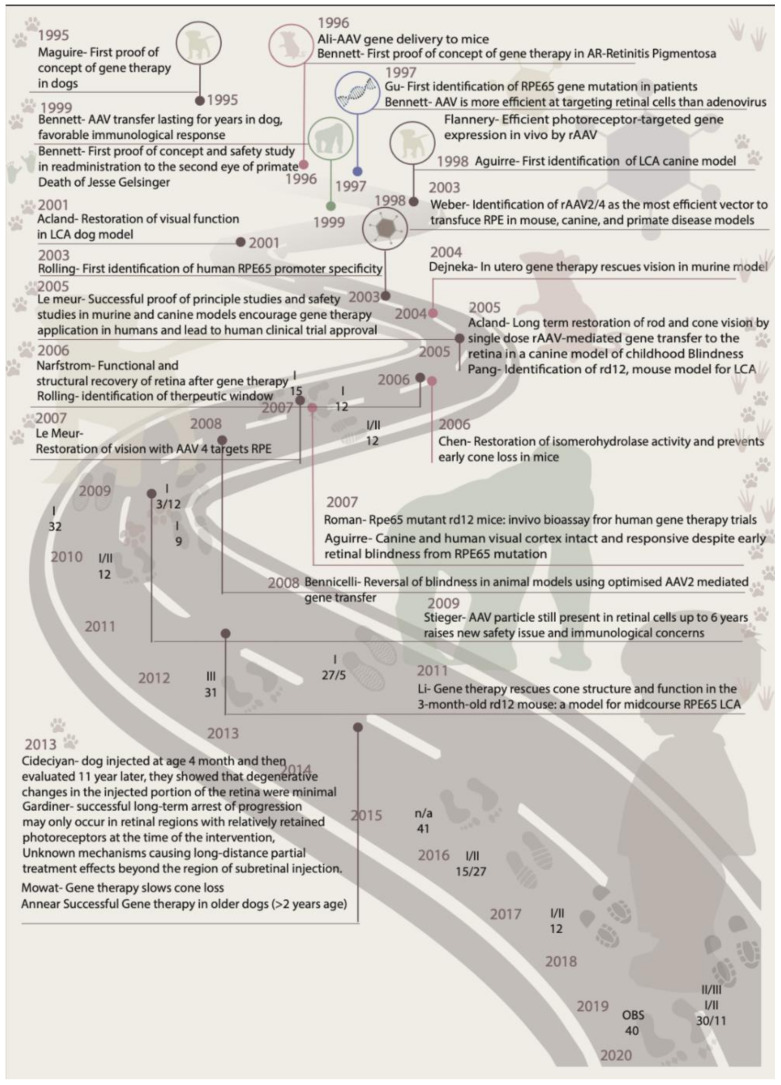
Preclinical milestones of establishing the *RPE65*-based gene therapy.

The role of *RPE65* mutations in the etiology of LCA was first identified in 1997 [45]. The dog model of *RPE65*-mutated LCA was established in 1998 [46], and was used for preclinical studies in 2001 [47]. The first clinical trial in humans was carried out in 2007 [5,48]. In a 2009 follow-up study, researchers identified the presence of AAV particles in primates from the study initiated six years ago, in 2003, raising safety issues and immunological concerns [49]. Together, these studies highlighted the need to identify possible roadblocks to clinical translation for the benefit of other gene therapies targeting retinal degeneration [50]. A roadmap of preclinical studies towards the success of RPE65-based gene therapy is shown in Figure 2.

## 5. Therapeutic Strategies and Clinical Milestones for LCA

Based on the establishments preclinical studies had achieved in gene augmentation therapy, clinical applications of various gene therapy strategies were carried out intercontinentally in the US along with University of London, Nantes University Hospital in France and Hadassah Medical Organization in Israel. Besides gene augmentation therapy that helps restore RPE65 by supplementing unmutated gene segments, other therapeutic strategies such as RNA-based antisense oligonucleotide therapy that reduce aberrant splicing and increase wildtype CEP290 production, 11-cis retinal therapy and gene editing therapy are as well in the run towards clinical feasibility (Table 2).

### 5.1. Gene Augmentation Therapy

**Figure 3:**
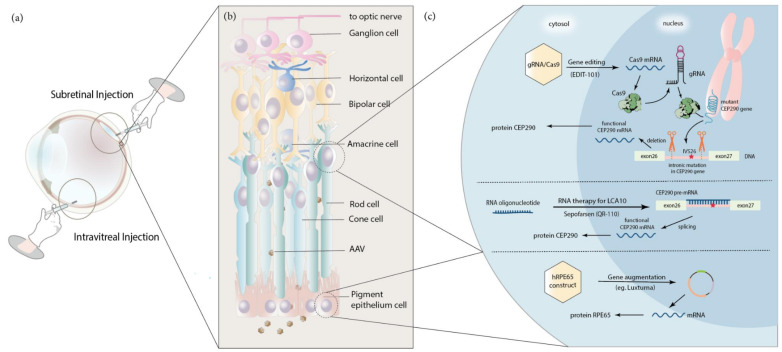
Overview of Leber Comgenital Amaurosis (LCA) retinal gene therapy. (**a**) Schematic illustration of subretinal injection and intravitreal injection. (**b**) Schematic of the retina structure showing the topology of retinal cell types. (**c**) The most well-established types of gene therapy of LCA include gene augmentation therapy (Luxturna), gene editing therapy (EDIT-101) and RNA antisense nucleotide-based therapy (QR-110). Top panel: gene editing therapy based on AAV-mediated subretinal delivery of CRISPR-Cas9 system that results in excision of intronic IVS26 mutation in the photoreceptor *CEP290* gene that causes abnormal splicing and termination of translation due to introduced cryptic exon. Middle panel: therapy based on intravitreal delivery of RNA antisense oligonucleotides targeting intronic mutation if *CEP290* gene that causes abnormal splicing. Oligonucleotides act at RNA level directing normal splicing of mutated pre-mRNAs. Bottom panel: gene augmentation aimed to correct the effect of the mutated *RPE65* in RPE is based on the delivery of the wild type *RPE65* gene by AAV vector to the subretinal area. Thus, normal RPE65 protein is partially restored, resulting in vision function improvement.

Since the first identification of the *RPE65* gene, researchers have successfully demonstrated safety and proof-of-principle studies using recombinant-AAV in LCA2 murine and canine models. These initial animal model studies encouraged further research in applying gene augmentation therapy in the human retina, which eventually led to human clinical trial approval [51]. Clinical trials by various groups began in 2007, including Spark Therapeutics in collaboration with the University of Pennsylvania testing their AAV2-hRPE65v2 vector, Applied Genetic Technology in collaboration with the University of Florida testing rAAV2-CB^SB^-hRPE65 and the NHS foundation trust in collaboration with the University of London testing rAAV2/2.hRPE65p.hRPE65. Moreover, by 2008, all three groups confirmed that the vector delivery was safe, and no adverse events were reported [5,48,52]. In 2011, a clinical phase I/II trial for the treatment of LCA2 patients started in France [53]. A schematic graph of how gene augmentation drug exerts it therapeutic effect is shown in Figure 3.

#### 5.1.1. Voretigene Neparvovec-Rzyl (Luxturna)

In 2007, Spark Therapeutics started the phase I trial of AAV2-hRPE65v2-based voretigene neparvovec-rzyl medication. Three months follow up showed that three patients with LCA2 demonstrated an acceptable safety profile with modest improvement in retinal function measured by subjective visual acuity tests. Although one patient developed an asymptomatic macular hole, which is an adverse event, the retinal function was nevertheless restored [48]. One year follow up showed that all patients exhibited a sustained improvement in measurements such as dark adaptometry, pupillometry, electroretinography, nystagmus and ambulatory behavior. A subgroup of children who gained ambulatory vision showed the most significant vision improvement [7].

Voretigene neparvovec-rzyl entered phase I/II in 2010. Consistently with the phase I trial, both safety and efficacy profile endpoints met expectations for at least 1.5 years. Furthermore, the subretinal injection of AAV2-hRPE65v2 was performed in three subjects, and the retinal function measurements persisted from 1 month to 1.5 years postinjection time points. The safety profile elicited no severe adverse events, although a transient rise in anti-AAV capsid antibodies in several patients was observed. The trial resulted in continuous function amelioration by showing improvements in velocity and amplitude of PLR, light sensitivity and reduction in nystagmus. The positive results in both safety and efficacy measurements further supported the usability of AAV-mediated gene augmentation therapy in treating *RPE65*-associated LCA [54].

In 2012, voretigene neparvovec-rzyl entered phase III and began to be administered into the contralateral eye. Previous exposure to the viral antigens may have prompted an immune response that could compromise the effect of the second administration. Concerned with safety, the team readministered the vector to the second eye of three adults at 1.7 to 3.3 years after the initial subretinal injection of AAV2-hRPE65v2. After six months, the evaluation of clinical examinations and immunological responses indicated the safety of interventions. In addition, the subjects showed improvements in visual/retinal function and cortical activation after the readministration of gene therapy [55]. AAV2-hRPE65v2 gene therapy successfully improved mean mobility and full-field light sensitivity after administration to the contralateral eye. To summarize, the evaluation of safety, immune response, retinal and visual functions and activation of the visual cortex, all supported the usability of AAV2-hRPE65v2 for gene augmentation therapy in *RPE65*-associated LCA [56].

Maximum visual and retinal function improvements were observed at 6 months post-treatment, and the improvement increased persistently throughout the trial [57]. Voretigene neparvovec-rzyl gene augmentation was shown to improve the visual function in *RPE65*-mediated LCA, with only two intervention participants having shown severe adverse events unrelated to the treatment, with other adverse events being mild [8]. The drug entered the final phase of clinical trials in 2019 with the observation phase. The completion of phase III trials made voretigene neparvovec-rzyl the first FDA-approved gene therapy for a genetic disease now sold under the commercial name Luxturna.

#### 5.1.2. tgAAG76

In 2007, University College London (UCL) in collaboration with Moorfields Eye Hospital NHS Foundation Trust initiated a phase I/II trial to test their product, rAAV2/2.hRPE65p.hRPE65 (tgAAG76). The result demonstrated that three young adult patients receiving subretinal injections developed no serious adverse effects. The evaluation revealed that tgAAG76 delivery did not result in any significant changes in retinal functions assessed by objective measurements. However, one patient significantly improved visual mobility and visual function on microperimetry and dark-adapted perimetry. One of the participants showed increased sensitivity to light and improved in an obstacle course under dim light. In summary, only very modest and temporary improvements in retinal sensitivity were observed rather than robust and durable therapeutic effects [5].

In 2016, the UCL team modified the rAAV2/2 vector to enhance RPE65 production efficiency without exceeding the tolerated vector dose to tackle the problem of possible intraocular inflammation. The modified vector, named AAV2/5-OPTIRPE65, contained an optimized promoter sequence, exogenous intron, optimized Kozak sequence, as well as codon optimization of the *RPE65* sequence. The AAV2/5 vector was used due to its superior gene delivery effectiveness to human RPE cells than the AAV2/2 vector [58]. The UCL team then successfully carried this modified vector into phase I/II in 2016.

#### 5.1.3. rAAV2-CB^SB^-hRPE65

The phase I trial of the University of Florida’s rAAV2-CB^SB^-hRPE65 began in 2007, whereby three young adults aged 21-24 years were recruited and received uniocular subretinal injections. Follow up lasted for 90 days, and the therapy passed safety requirements since no severe adverse events or systemic toxicity were detected. The participants showed limited visual acuity improvement, yet they all reported that the treated eye had better visual sensitivity than the contralateral eye, especially under dim light [52]. The measurements at 2 weeks, 3 months and 1 year time points indicated that rAAV2-CB^SB^-hRPE65 augmentation did not elicit vector-related serious adverse events, and those significant improvements in visual sensitivity reported at the 3 month-time point extended to 12 months. The safety and efficacy of rAAV2-RPE65 gene therapy could last at least one year post-treatment [59]. There was no systemic toxicity reported for safety concerns, while ocular adverse events were due to surgery [60]. Until 2015, the clinical trials indicated that visual gain was detectable within the first month and efficacy could persist for at least three years. Longer follow-up observations found that although the treated eye’s improved vision persisted, there was progressive photoreceptor degeneration which caused a diminution of visual sensitivity [6].

#### 5.1.4. rAAV2/4-hRPE65

rAAV2/4-hRPE65 underwent a phase 1/2 trial in 2011 conducted by the University of Nantes, and the results were reported in 2018. All patients showed good immune tolerance to the vector with improved visual acuity, visual field and cortical activations along visual pathways [10]. In this study, patients were segregated into cohorts based on the age and dose of the viral vector injected. Le Meur et al. identified that, compared with previous clinical trials of other vectors, the dosage designed for the rAAV2/4 vector (1.22 × 10^10^ vg to 4.8 × 10^10^ vg) was lower than that for vectors from other studies (ranging from 1.5 × 10^10^ vg to 6.11 × 10^11^ vg), indicative that in haplodeficient diseases, the designed dosage may have been insufficient to restore enough functional protein to slow down the degeneration process and improve visual function.

### 5.2. 11-Cis-Retinal Replacement: QLT091001

QLT091001, a synthetic 9-cis-retinyl acetate, is an oral drug that serves as a replacement for the missing 11-cis-retinal, which combines with opsin to form its photoactive form required for the phototransduction cascade. The chromophore reaches the retina in a non-invasive manner and can be withdrawn if necessary. It enables bypassing through the block of the retinoid cycle and helps to preserve the morphology of the retina. The administration of QLT091001 was shown to improve visual function as measured by Goldmann visual field, visual acuity and functional MRI; positive subjective observations were also reported by patients [58]. QLT091001 entered a phase I trial enrolling 32 patients in 2009. The authors claimed that non-invasive oral therapy is well tolerated, and it improved the visual functions of LCA patients with RPE65 and LRAT defects. Scholl et al. used high-definition optical coherence tomography and found a positive correlation of the photoreceptor layer’s larger baseline OS thickness with treatment response prediction [61].

### 5.3. RNA-Based Antisense Oligonucleotide Therapy: QR-110 (Sepofarsen)

Unlike LCA2 driven by the defective RPE65 enzyme, LCA10 is associated with splicing defects in cilia transport protein CEP290. LCA10 patients’ *CEP290* pre-mRNA contains a hypomorphic cryptic splice site in intron 26 [62,63,64,65]. As a result, two *CEP290* transcripts are produced: wild type transcripts are produced in parallel with alternatively spliced transcripts containing an extra cryptic exon of 128 nucleotides that introduces a premature stop codon (p.Cys998*). The c.2991 + 1655A > G allele’s hypomorphic nature results in significantly lower levels of functional CEP290, a key protein of the primary cilia [66,67,68]. Photoreceptors are particularly vulnerable to disruptions of cilia function [69]. QR-110 is a single-stranded 2′-O-methylated RNA antisense oligonucleotide that corrects splicing defect arising from the *CEP290* c.2991 + 1655A > G mutation. (Figure 3) QR-110 was shown to restore the normal splicing of *CEP290* in homozygous retinal organoids carrying the *CEP290* c.2991 + 1655A > G mutation and thus restore normal *CEP290* mRNA and protein, leading to increased ciliogenesis. Moreover, QR-110 was proven to be effective when used on retinal organoids due to its good accessibility to the retina and good tolerance following intravitreal (IVT) injection in humans [70]. QR-110 entered phase II in 2017, followed by phase II/III in 2019. There were no serious adverse events, and visual acuity, self-reported clarity and brightness in the treated eye were encouraging for the application of QR-110 in LCA10 patients.

### 5.4. Gene Editing Therapy: EDIT-101

The collaborative effort of Allergan, Editas Medicine and the University of Florida translated the very first gene-editing approach to treat LCA10 to clinical trials in 2019. AGN-151587 (EDIT-101) was designed to excise the pathogenic splice site, which would result in the normal splicing and expression of CEP290 protein [71]. (Figure 3.) Another group using a single AAV vector, Maeder et al., circumvented the limitation of the carrying capacity of AAV by using a smaller *Staphylococcus aureus* CRISPR (Clustered regularly interspaced short palindromic repeats)-Cas9 system that allowed an AAV5 vector to deliver the Cas9 gene and two guide RNAs [72] under the regulation of the *GRK1* promoter that is specific to photoreceptors only. Advance in preclinical study was shown as EDIT-101 performed gene editing after its delivery to humanized CEP290 mice. Hence, in September 2019, EDIT-101 entered clinical trial phase I/II with 19 patients enrolled to evaluate single ascending dose effects in participants with LCA10. To date, no study report has been published.

## 6. Limitations and Challenges

A comprehensive review of clinical trial reports brought to light numerous unmet challenges of retinal viral gene therapy. These include the lack of a control vector to distinguish the therapeutic effect between the vector and RPE65 re-establishment to ensure that any improvements specifically reflect the expression of the protein encoded in the AAV vector. Therapy complications generally do not arise from exposure to viral vectors but rather from intervention with the subretinal administration. Furthermore, high patient success variability and a lack of reliable baseline comparison within a small sample group raise concerns on upgrading/refining future development.

### 6.1. Surgical Complications

In 1969, Young and Bok identified that primate photoreceptors renew their OS within two months [73]. In 1993, it was shown that the standard configuration of the experimentally detached retina returned to normal within 1 to 2 months [74], laying the foundations for the use of the subretinal intervention. However, it was not until 2000 that Cooper and Thomas consolidated the use of subretinal surgery techniques [75]. Due to the limited target effect of intravitreal delivery in the posterior segment of the retina, only subretinal delivery of vectors can ensure safe and efficient transduction of photoreceptors and RPE cells [6]. The most common AAV vectors do not efficiently target the outer retina following intravitreal injection [76,77,78,79]. While most postoperative subretinal detachments are reversible and subject to repair, several studies reported myriad surgical complications that may result in macular hole development [48,54,60]. These common complications include effusions, hypotonia, retinal tears, subconjunctival hemorrhage, ocular hyperemia and increased intraocular pressure [52,60,80]. Bainbridge et al. devised a safer administration, whereby up to 1mL of rAAV vector could be delivered through a single retinotomy without causing tears in the thinned, degenerated retina [81]. With the complications delineated above, subretinal injections are thus contraindicated in fragile and degenerating retinas, significantly limiting the population who may benefit from the therapy. The demand for intravitreal injection has ignited research on the directed evolution or design of specific capsid mutations that may lead vectors to target the outer retina via intravitreal injection [82,83]. Furthermore, subretinal injection is a procedure that requires a surgical room and general anesthesia that also possesses inherent risks. Surgical complications necessitate the development of vectors that may target the outer retina via intravitreal injection, which may also be performed at a clinic with local anesthesia.

### 6.2. Variability of Patient Response

Patient response to *RPE65* gene therapy was shown to be variable within and between studies. Weleber et al. observed the gain of visual function in 9 out of 12 patients. However, the individual visual function parameters that improved varied among these patients, and two patients showed a decrease in BCVA in the treated eye compared to the untreated eye [80]. In contrast to the successful stories exemplified by Luxturna, the trial (NCT00821340) testing the rAAV2-hRPE65 vector showed drastically different results. Instead of improving visual acuity, post-treatment visual acuity decreased predictably due to the temporal retinal detachment induced by subretinal administration and returned to preoperative levels only by six months [48]. No clinically significant improvement was observed in any of the three patients, as determined by Goldmann perimetry testing, microperimetry, or pattern electroretinography. The advertised advantage of *RPE65* gene therapy was the improvement of nystagmus; however, nystagmus in two patients who suffered from high-amplitude nystagmus showed no resolution [48]. This was consistent with a trial by Cideciyan, where it was reported that none of their patients demonstrated decreases in nystagmus. Out of the three patients, visual function only improved in one patient [48]. The varied patient response may be a consequence of a considerable variation in baseline visual function and differences in vector administration site [80].

Analyzing the physiology behind it, Cideciyan proposed two hypotheses for slow chromophore delivery rate: (i) reduced rate of synthesis and (ii) increased physical or chemical barrier to its transport. The evidence supporting the second hypothesis includes: (1) the dramatic accumulation of all-trans-retinyl ester (precursor of rhodopsin) in RPE, (2) disorganized and unhealthy rod OS that aggravates the resistive barrier [46,84,85,86] and (3) altered RPE/photoreceptor interface due to subretinal administration induced retinal detachment [87]. The low expression of *RPE65* despite successful retinal transfection could reduce the rate of chromophore synthesis and act as an enzymatic limiting stage to rhodopsin regeneration, as observed in mice engineered to express low levels of wild type *RPE65*. This enzymatic bottleneck must be circumvented with a vector that allows the higher expression of *RPE65* [88].

Furthermore, limitations of the small sample of patients are aggravated when the study design does not involve the randomization of eyes, namely, clinicians tend to preferentially treat patients with worse vision. Another bias arises when examiners are not masked to the study eye versus control eye. The statistical approach was thus confounded by numerous issues [60]. During the postoperative period, patients may have an inclination to use the control eye and are unaware of visual gain in the treated eye, unless dictated to do so [60]. An additional confounder arises from the presence of nystagmus and amblyopia. These conditions would dictate the amount of time the eye can fixate on a particular object and is known to affect the degree of cortical activation that may induce false positive improvements in LCA patients [89]. Lastly, the heterogeneity of disease progression and the nature of *RPE65* mutation should be evaluated and grouped on an individual basis for relevant comparisons [60].

### 6.3. Readministration Safety

Voretigene neparvovec-rzyl was the first randomized gene therapy trial for a genetic disease to enter phase 3. Russel et al. stress that the current clinical trials that use an uninjected contralateral eye as a control are not reflective of the clinical scenario where bilateral administration would be given. Unilateral injection of therapeutics also does not allow the relevant assessment of systemic effects [8]. Hence, Bennett et al. aimed to address whether the readministration of virus-mediated gene delivery to the patients exposed to priory delivery with the same vector may elicit immune responses that would diminish the retinal improvements and visual function of the originally injected eye. An intriguing observation revealed that the initially injected eye’s function improved after the readministration into the second eye, as shown by fMRI. Surprisingly, the second treated eyes had better clinical performance than the first treated eye, despite being severely damaged for more than 2.5 decades. After confirmation by pupillometry, fMRI and full-field sensitivity testing, the second eye became more sensitive to dim light. A universal phenomenon observed in numerous trials was that post-treatment retinal and cortical responses did not show spontaneous recovery. Similar to experimental results in large animals, the progression in retinal and cortical responses showed a continuous increase in early stages and plateaued between 1.5 and 3 months postadministration [55]. Noteworthy, after confirming the therapeutic effects and safety in three adults who had received a unilateral subretinal injection of AAV2-hRPE65v2 previously, Bennet et al. aimed to restore vision in injected eyes and evaluate the safety and efficacy of the second injection in the NCT01208389 trial [55]. As the AAV capsid and foreign *RPE65* are potential antigens, physicians were concerned that the readministration would result in an inflammatory response upon re-exposure. Nevertheless, Bennet et al. demonstrated that readministration was not accompanied by the potentially damaging immune response. The safety profile of readministration may be due to the eye’s immune-privilege nature and the small volume of the ocular compartment that enables a low vector dosage [55].

### 6.4. Age-Dependent Outcome Controversy

Bennett et al. also observed that younger individuals tended to achieve better vision recovery than older patients. However, these results may also reflect an age effect, whereby the retina from younger patients had not undergone as much deterioration and toxin buildup [55]. Nevertheless, Jacobson et al. argued that age did not significantly affect either the overall visual improvement parameters of the participants. Surprisingly, the most dramatic visual acuity increases were observed in two older patients (aged 24 and 30). Jacobson et al. further addressed the reason behind the proposed trend as a matter of emphasis. They believed a similar improvement in a child patient’s light sensitivity would be emphasized over the same improvement in an adult patient. Similarly, the younger patients’ mobility performance was studied uniocularly with lower room illuminations, whereas the older patients were studied binocularly at 250 lux light level [60]. An ongoing trial by Maguire et al. aimed to determine whether treatment efficacy may be enhanced in a population with amblyopia and retinal degeneration has not been concluded [48].

### 6.5. Inappropriate Clinical Markers

It is not clear whether the improvement in visual responses is entirely due to enhanced levels of RPE65 in the retina. It is not safe and ethical to obtain evidence merely through the biopsy of retinal material [5]. Some studies have demonstrated improved navigational abilities using the newly injected eye [55]. Functional assays to study retinal function, such as navigational abilities, were elaborated through trial and error. Ashtari et al. therein pioneered using more objective parameters, such as psychophysical testing and fMRI measurements, to study the responsiveness or activation of neurons in the visual cortex that processes vision. Their study confirmed that the gene augmentation therapy of LCA2 patients rendered the retina (and visual cortex) activation more sensitive to dimmer light and lower contrast stimuli [89]. Maguire et al. confirmed an increase in visual acuity post-treatment when comparing low levels of spatial vision to recovery levels that were still low. The improvement of vision in dim light was patient self-reported and not objectively quantified [48]. Since the clinical presentation, the hallmark of LCA is nyctalopia, night blindness that makes navigation difficult in dim light. Hence, the novel outcome measure of functional vision based on assessing different degrees/Lux of light levels and navigation accuracy, called the Multi-luminance Mobility Test (MLMT), was proposed. The MLMT measures individuals’ navigating while avoiding obstacles at seven standardized illumination levels with a pass or fail based on course navigation completed within 180 s with three or fewer errors. Additionally, a positive score indicates passing the MLMT at a lower light level.

### 6.6. Short Follow-Up and Small Sample Trial

Bennett et al. maintained a follow up of six months, but acknowledged the need for longer follow up and a larger patient pool to determine safety and efficacy measures and to identify factors influencing the extent and duration of visual recovery [55]. To circumvent the small sample size bias, Russell et al. designed a measurement method that allows statistical high-power acquisition for a small trial. Using the MLMT framework to convert various lighting conditions into a continuous metric provides the quantification of the continuous improvement [8].

### 6.7. Undeterred Onset of Photoreceptor Degeneration

Gene augmentation therapy for RPE65 deficiency using AAV2/2 vectors can improve different aspects of sight in humans [5,48,52,80,90]. However, the central question in gene therapy efficacy is whether the disease progression can be halted. Progressive retinal degeneration limits the efficacy of retinal sensitivity in the long run, and the functional improvements in photoreceptor cells found in animal models have been relatively mild in humans [6,81,91]. Yet, subjective outcome measurements, such as visual acuity, Goldmann perimetry, navigational vision and pupillometry, were reported to reveal improvements. RPE65 protein expression in humans is greater than in dogs, indicating a greater demand for RPE65 protein in the former. The balance between efficacy and dosage-induced toxicity limits the benefits of current vectors [92]

Young RPE65-mutant dogs (4 months) were given interventions and then evaluated 5–11 years later. Minimal degenerative changes in the injection sites indicated that gene therapy indeed halted degeneration progress. However, such beneficial effects were not observed in humans [93]. This raises questions as to whether variations in age, stage of disease, species, mutations or complications cause the difference. Treatment in older age may not prevent structural degeneration. Despite the uncertainties, phase I trials have shown that gene therapy is safe, stable and restores partial retinal function [94].

## 7. CRISPR-Cas9 System Application in Inherited Retinal Dystrophies

Gene augmentation therapy through AAV-mediated gene delivery has shown promising therapeutic effects clinically; however, the stringent cargo limit of vectors narrows its application to only a portion of cases with mutations in causative genes of relatively small size [95]. Thus, approaches other than gene augmentation are in demand for a vast population of IRD patients. CRISPR-Cas9 is emerging as a new gene-editing tool to address the issue. The system utilizes guide RNA (gRNA) to direct Cas9 endonuclease to specific gene loci proximal to a protospacer adjacent motif (PAM), inducing a double-strand break (DSB). Cell machinery then repairs the CRISPR-targeted site by performing non-homologous end joining (NHEJ) or homology-directed repair (HDR) [96,97,98].

To date, retinal gene therapy approaches can be categorized into mutation dependent and mutation independent. In the mutation-independent approach, all endogenous chromosomal target genes, namely, both of the alleles, are ablated permanently by the CRISPR-Cas9 technique, while exogenous wild type cDNA is supplemented to complement the gene loss. P23H and D190N are two common mutations in the rhodopsin (*RHO*) gene that result in the autosomal dominant retinitis pigmentosa (ADRP) [99]. Two separate vectors, AAV-gRNA-mRho promoter-hRHO cDNA and AAV-Cas9, were mixed and subretinally injected into a RhoP23H or RhoD190N mutation knock-in mouse model. The ablate and supplement treatment caused an improvement in retinal outer nuclear layer thickness and better preservation of a and b wave amplitude compared to the gene augmentation control only group [100]. Koji M. et al. proposed a system termed microhomology-mediated end joining (MMEJ), with small microhomology arms of approximately 20 bps, MMEJ allows the specific integration of complementary DNA into the desired genome loci via a single AAV platform. Compared with dual vector-based mutation replacement, the MMEJ system showed an improvement in genome editing efficacy and thus showed robust restoration of the visual function in retinal dystrophy mouse models [101]. The combination of CRISPR-Cas9-NHEJ or MMEJ that mediated mutant allele knockout and concomitant delivery of the wild type cDNA that compensated for the loss of the endogenous target gene combination has shown potency to correct mutations via AAV-mediated delivery.

In a mutation-dependent approach, dominant mutant genes in the heterozygous pair of alleles responsible for the dysfunction can be selectively disrupted, while the wild type allele gene should maintain its expression. Differentiation between wild type and mutant alleles during Cas9 cleavage is achieved through the requirement of Cas9 activity upon recognizing PAM. Serine amino acid substitution at the 334/338 site results in the dysfunction of rhodopsin protein and affects protein trafficking to the photoreceptor OS. The selective deletion of *Rho*^S334^ in the rat model stopped the RHO^S334^ production; meanwhile, normal *Rho* could be expressed dominantly [102]. Another example of mutant knockout was demonstrated in the retina of RhoP23H heterozygous mice. Results showed a reduced expression of mutant P23H transcript and improved thickness of photoreceptor layers [103].

Though the CRISPR-Cas9 system has revolutionized gene therapy in several aspects, the drawbacks are that CRISPR mandates highly allele-specific designs, while gene defects underlying IRDs are extremely heterogeneous. Genetic heterogeneity has made the development of IRD gene therapy prohibitive, as clinical trials are mandatory for every mutation [104]. As a result, instead of manipulating the mutant genes, some studies turned to targets that could be applied to a larger population, for example, the neural retina-specific leucine zipper (*Nrl*) gene. In RP disease progression, the loss of cone, which results in blindness, is considered to be a secondary effect of rods degeneration. The *Nrl* gene is a determinant in the development of rod photoreceptors, and it also plays a key role in maintaining rod function and homeostasis in mature rod photoreceptors. Consequently, the knockdown *Nrl* by AAV-mediated CRISPR-Cas9 in mice induced morphology and functional changes in rods, allowing the cells to overcome the degeneration progress [105].

## 8. Conclusions and Future Initiatives

In summary, the evolution of gene therapy has overcome many barriers to achieve translation from fundamental science advancements to clinical development and proved to be relatively safe in multiple clinical trials, albeit with variations in outcomes. Among these clinical trials, voretigene neparvovec (Luxturna) that targets *RPE65*-associated LCA was the first FDA-approved gene therapy drug, while other clinical trials of IRDs, including Retinitis Pigmentosa (RP), Choroideremia and X-linked Retinitis Pigmentosa (XLRP), are also under way. In addition to Luxturna, three clinical trials advanced to Phase III collectively, including antisense oligonucleotides-based QR-110 for LCA10, AAV5-RPRG gene-drug for XLRP and AAV2-REP1 for choroideremia (Table 1). To date, clinical trials are mostly gene augmentation therapies that deliver the correct gene to the retina to restore its function. A few studies have taken advantage of antisense oligonucleotides to direct the normal splicing of mutant pre-mRNA and thus correct the mutation on the mRNA level. A gene editing strategy using the CRISPR-Cas9 system is utilized in correcting gene aberrations by editing the pathogenic variants in a targeted fashion. Despite the advances in IRD gene therapies, how to tackle IRDs presenting with early onset degeneration or causing aberrant retinal development is unsolved. In such cases, gene augmentation intervention may be applied early, even in utero, raising ethical and practical challenges. Furthermore, AAV-based gene therapy drawbacks include low transduction efficiency and viral capsule immunogenic adverse effects; thus, proper dosage optimization is required to find a compromise between these two factors. Each RPE cell serves up to 40 photoreceptor cells in the primate retina [106]; therefore, targeting RPE-specific mutations (such as *RPE65*) results in an amplified intervention effect and requires a lower dosage. On the other hand, targeting photoreceptor-specific genes may require the optimization of vectors to increase the tropism towards this cell type. As such, AAV8-based vectors can be more preferential than AAV2-based vectors, as the former is more efficient in transducing photoreceptors, to date; the development of higher efficiency viral vectors may be required in the future [107].

## Figures and Tables

**Table 1:** Update of clinical trials of gene therapies in inherited retinal dystrophies.

Gene Target	Phase	Enrolled #	Start Date	Est. End Date	Drug/Vector	Sponsor	Trial Number	Ref.
Leber Congenital Amaurosis								
RPE65	I/II	12	Jan-2007	Dec-2014	tgAAG76 (rAAV2/2.hRPE65p.hRPE65)	University College London	NCT00643747	[5]
	I	15	Jul-2007	Jun-2026	rAAV2-CB^SB^-hRPE65	University of Pennsylvania	NCT00481546	[6]
	I	12	Sep-2007	Mar-2018	voretigene neparvovec-rzyl (AAV2-hRPE65v2)	Spark Therapeutics	NCT00516477	[7]
	I/II	12	Nov-2010	Nov-2026			NCT00999609	[8]
	III	31	Oct-2012	Jul-2029			NCT01208389	
	I/II	12	Jun-2009	Sep-2017	rAAV2-CB^SB^-hRPE65	Applied Genetic Technologies Corp	NCT00749957	[9]
	I	3	Feb-2009	Jan-2017	rAAV2-hRPE65	Hadassah Medical Organization	NCT00821340	
	I/II	9	Sep-2011	Aug-2014	rAAV-2/4.hRPE65	Nantes University Hospital	NCT01496040	[10]
	I/II	15	Apr-2016	Dec-2018	AAV2/5-OPTIRPE65	MeiraGTx UK II Ltd.	NCT02781480	[11]
	I/II	27	Nov-2016	Apr-2023			NCT02946879	
CEP290 p.Cys998X	I/II	11	Oct-2017	Oct-2019	QR-110 (Antisense oligonucleotides)	ProQR Therapeutics	NCT03140969	
	I/II	11	May-2019	Mar-2021			NCT03913130	[12]
	II/III	36	Apr-2019	Dec-2021			NCT03913143	
CEP290 Intron 26 (IVS26)	I/II	18	Sep-2019	Mar-2024	EDIT-101 (CRISPR-Cas9)	Editas Medicine, Inc.	NCT03872479	
Advanced Retinitis Pigmentosa								
ChR2	I/II	14	Dec-2015	Apr-2035	RST-001	Allergan	NCT02556736	
Retinitis Pigmentosa (RP)								
PDE6B	I/II	15	Nov-2017	Sep-2024	AAV2/5-hPDE6B	Horama S.A.	NCT03328130	
RLBP1	I/II	21	Aug-2018	Jul-2026	CPK850	Novartis Pharmaceuticals	NCT03374657	
USH2A	I/II	18	Mar-2019	Jun-2022	QR-421a	ProQR Therapeutics	NCT03780257	
PDE6A	I/II	9	Sep-2019	Dec-2025	rAAV.hPDE6A	STZ eyetrial	NCT04611503	
Autosomal Dominant Retinitis Pigmentosa								
RHO	I/II	35	Oct-2019	7-Oct-2021	QR-1123	ProQR Therapeutics	NCT04123626	
X-linked Retinitis Pigmentosa (XLRP)								
RPGR	I/II	37	Jun-2020	Sep-2023	4D-125	4D Molecular Therapeutics	NCT04517149	
	I/II	50	Mar-2017	Nov-2020	BIIB112	NightstaRx Ltd., a Biogen Company	NCT03116113	[13]
	I/II	46	1Jul-2017	Nov-2020	AAV2/5-RPGR	MeiraGTx UK II Ltd.	NCT03252847	
	III	48	Jan-2021	Jul-2022	AAV5-RPRG	MeiraGTx UK II Ltd.	NCT04671433	
	I/II	42	1Apr-2018	Aug-2026	AGTC-501 (rAAV2tYF-GRK1-RPGR)	Applied Genetic Technologies Corp	NCT03316560	
Choroideremia								
REP1	I/II	14	Oct-2011	Oct-2017	rAAV2.REP1	University of Oxford	NCT01461213	[14]
	I/II	6	Apr-2015	Sep-2025	rAAV2.REP1	University of Alberta	NCT02077361	
	II	6	Sep-2015	Feb-2018	rAAV2.REP1	University of Miami	NCT02553135	[15]
	II	6	Jan-2016	Feb-2018	rAAV2.REP1	STZ eyetrial	NCT02671539	[16]
	II	30	Aug-2016	Aug-2021	AAV2.REP1	University of Oxford	NCT02407678	
	III	170	Dec-2017	Dec-2020	AAV2-REP1	NightstaRx Ltd., a Biogen Company	NCT03496012	[17]
	II	60	Nov-2018	Feb-2022	BIIB111(AAV2-REP1)	NightstaRx Ltd., a Biogen Company	NCT03507686	
CHM	I/II	15	Jan-2015	Oct-2022	AAV2-hCHM	Spark Therapeutics	NCT02341807	
	I	15	Jul-2020	May-2023	4D-100	4D Molecular Therapeutics	NCT04483440	
X-linked juvenile retinoschisis								
RS1	I/II	24	Feb-2015	Jul-2023	AAV8-scRS/IRBPhRS	National Eye Institute (NEI)	NCT02317887	
	I/II	27	May-2015	Oct-2023	rAAV2tYF-CB-hRS1	Applied Genetic Technologies Corp	NCT02416622	

RPE65: Retinal pigmented epithelium-specific protein with molecular mass 65 kDa; CEP290: Centrosomal Protein 290; ChR2: Channelrhodopsin-2; PDE6B: Phosphodiesterase 6B; RLBP1: Retinaldehyde-binding protein 1; USH2A: Usherin; PDE6A: Phosphodiesterase 6A; RHO: Rhodopsin; RPGR: Retinitis pigmentosa GTPase regulator; REP1: Rab escort protein 1; CHM: Choroideremia; RS1: Retinoschisin.

**Table 2:** Summary of therapeutic strategies for Leber Congenital Amaurosis.

	University	Drug	Company	Genome	Capsid	Gene Modifications	Promoter	Polyadenylation	Dosage	Administration
Gene Augmentation Therapy	Pennsylvania	Luxturna	Sparks Therapeutics Inc.	2	2	modified Kozak sequence/hybrid cytomegalovirus enhancer	β-actin promoter	SV40	1.5E10 to1.5E11 vg	Single-dose subretinal injection
	London	tgAAG76	Targeted Genetics Corp	2	2		hRPE65	BSA	1E11	Single-dose subretinal injection
	London	OPTIRPE65	MeiraGTx UK II Ltd.	2	5	Condon-optimized/ Kozak sequence	NA65p Optimized hRPE65 promoter	SV40	-	Single-dose subretinal injection
	Florida	rAAV2-CB^SB^-hRPE65	National Eye Institute, AGTC	2	2	CMV enhancer	CB^SB^ Cystathionine-beta-synthase		5.96E10 to 18E10	Single dose subretinal injection
	Nantes	rAAV2/4- hRPE65	AFM, Horama	2	4	-	hRPE65	BSA	1.2E10 to 4.8E10	Single-dose subretinal injection
	Israel	rAAV2-CB-hRPE65	Hadassah Medical Organization	2	2	CMV enhanced	β-actin promoter		1.19E10	Single-dose subretinal injection
	Oregon	rAAV2-CB-hRPE65	Applied Genetic Technology	2	2	CMV enhanced	β-actin promoter		1.8E11 to 6E11	Single-dose subretinal injection
	University	Drug	Company	Component					Dosage	Administration
11-cis Retinal Replacement	Illinois	QLT091001	QLT Inc.	synthetic 9-cis-retinyl acetate					40 mg/m^2^/day	7-day oral administration
	University	Drug	Company	Component					Dosage	Administration
RNA Therapy	Iowa	QR-110 Sepofarsen	ProQR Therapeutics	2′ O-methyl-modified RNA oligonucleotide					-	intravitreal injection
	University	Drug	Company	Capsid	g-RNA/Cas9	Gene modifications	Promoter	Polyadenylation	Dosage	Administration
Gene Editing Therapy	Florida	EDIT-101	Allergan	5	CEP290-323 & CEP290-64/Staphylococcus aureus Cas9	consensus kozak sequence and ATG start codon	human U6 polymerase III	SV40	-	Single-dose subretinal injection

## Abbreviations

AAV	Adeno-associated virus
ADRP	Autosomal dominant Retinitis Pigmentosa
CEP290	Ciliopathy gene centrosomal protein 290
CHM	Choroideremia
CRISPR	Clustered regularly interspaced short palindromic repeats
DSB	Double strand break
GPCR	G-protein coupled receptor
gRNA	Guide RNA
HDR	Homology-directed repair
IRBP	Interphotoreceptor retinoid-binding protein
IRDs	Inherited Retinal Dystrophies
IVT	Intravitreal injection
LCA	Leber’s Congenital Amaurosis
LRAT	Lecithin retinol acyltransferase
MMEJ	Microhomology-mediated end joining
NHEJ	Non-homologous end joining
Nrl	Neural retina-specific leucine zipper protein
RHO	Rhodopsin
RP	Retinitis Pigmentosa
RPE	Retinal pigment epithelium
rAAV	Recombinant Adeno-associated virus
O.S.	Outer segments
PAM	Protospacer adjacent motif
XLRs	X-linked juvenile retinoschisis
